# Auranofin and iodoquinol as promising repurposing drugs against filamentous fungi: antifungal activity and cellular alterations

**DOI:** 10.1128/spectrum.03356-25

**Published:** 2026-02-27

**Authors:** Mariana Ingrid Dutra da Silva Xisto, Rodrigo Rollin-Pinheiro, Paloma Cristina Malfetano da Rosa, Johnatha de Souza Santos, Nicole Ferreira da Silva Irmão, Julia Almeida Abi Abib, Victor Pereira Rochetti, Yuri de Castro Almeida, Giulia Maria Pires dos Santos Freitas, Jefferson Cypriano, Eliana Barreto-Bergter

**Affiliations:** 1 Laboratório de Química Biológica de Microrganismos, Departamento de Microbiologia Geral, Instituto de Microbiologia Paulo de Góes, Universidade Federal do Rio de Janeiro (UFRJ) 28125, Rio de Janeiro, Brazil; 2 Departamento de Microbiologia, Imunologia e Parasitologia, Faculdade de Ciências Médicas, Universidade do Estado do Rio de Janeiro (UERJ) 28130, Rio de Janeiro, Brazil; 3 Unimicro, Departamento de Microbiologia Geral, Instituto de Microbiologia Paulo de Góes, Universidade Federal do Rio de Janeiro (UFRJ) 28125, Rio de Janeiro, Brazil; University of Florida College of Dentistry, Gainesville, Florida, USA

**Keywords:** auranofin, iodoquinol, fungal infections, repurposing drugs, antifungal therapy

## Abstract

Opportunistic fungal infections have become an increasing health threat, particularly in immunocompromised individuals, due to their high virulence, biofilm-forming capacity, and resistance to conventional antifungals. Given the limited therapeutic arsenal, drug repurposing represents a promising strategy for antifungal discovery. This study evaluated the antifungal activity and mechanisms of auranofin and iodoquinol against filamentous fungi listed as World Health Organization (WHO) priority pathogens, including *Aspergillus fumigatus*, *Fusarium oxysporum*, *Scedosporium boydii*, *Lomentospora prolificans*, *Rhizopus oryzae*, *Mucor velutinosus*, and *Cunninghamella* sp. Although *Aspergillus flavus* is not included in the WHO priority list, it was included in this study due to its clinical relevance as a major cause of aspergillosis and fungal rhinosinusitis. Auranofin displayed fungicidal activity against *Aspergillus* spp., *L. prolificans*, *S. boydii*, and *R. oryzae*, while iodoquinol exhibited potent but mainly fungistatic effects, with minimum inhibitory concentration values ranging from 0.625 to 20 µM. Both compounds inhibited early growth and reduced preformed biofilms by over 50% in biomass and metabolic activity. Stress susceptibility and fluorescence assays indicated that auranofin interferes with lipid homeostasis, cell wall integrity, and oxidative stress responses, whereas iodoquinol alters membrane lipids, mannose, and chitin distribution, suggesting multitarget surface effects. Scanning electron microscopy revealed consistent ultrastructural alterations, including hyphal deformation and increased extracellular matrix deposition in *A. fumigatus* and *F. oxysporum* treated with sub-inhibitory drug concentrations, corroborating the observed cellular stress responses. Drug interaction assays demonstrated additive or synergistic effects with voriconazole and amphotericin B, but not with posaconazole. Overall, auranofin and iodoquinol exhibit broad-spectrum antifungal activity, disrupt fungal morphology and physiology, and represent promising repurposed candidates for developing novel combination therapies against refractory mycoses.

**IMPORTANCE:**

Mycoses caused by opportunistic fungi are an increasingly public health problem, particularly in immunocompromised individuals, due to their high virulence and resistance to conventional antifungals. The World Health Organization’s priority fungal pathogens list points out a variety of filamentous fungi, such as *Aspergillus fumigatus*, *A. flavus*, *Fusarium oxysporum*, *Scedosporium boydii*, *Lomentospora prolificans*, *Rhizopus oryzae*, *Mucor velutinosus*, and *Cunninghamella* sp. Given the limited therapeutic arsenal to combat them, drug repurposing represents a promising strategy for antifungal discovery. The present study evaluated the antifungal activity and some cellular alterations caused by auranofin and iodoquinol, which are already available in clinical settings to treat rheumatoid arthritis and infections by amoeba, respectively. The data presented in the study revealed that both drugs display a wide spectrum of action against different fungal pathogens, as well as contribute to highlight the potential of them as repurposing drugs to be investigated as alternatives for the treatment of mycoses.

## INTRODUCTION

Fungal infections have been emerging worldwide in recent decades, especially due to the increasing number of individuals presenting a base disease that serves as a risk factor, such as diabetes and some immunosuppressive conditions, including HIV/AIDS, organ transplants, and hematological illness. Currently, it is estimated that 1.6 million deaths are caused by pathogenic fungi annually (1). Among these pathogens, opportunistic filamentous fungi are highlighted due to their high mortality rates, and the most frequent genera found in clinics are *Aspergillus*, *Fusarium*, *Scedosporium/Lomentospora,* and the group of Mucorales (2). Recently, the COVID-19 pandemic has shown that these pathogens, especially Mucorales species, play relevant roles as causative agents of secondary infections in patients infected with SARS-CoV-2 (2).

A concerning factor that contributes to the high mortality rates displayed by these pathogens is the limited therapeutic options available in clinical settings. Voriconazole is the main choice to treat infections caused by *Aspergillus*, *Fusarium,* and *Scedosporium*/*Lomentospora*, whereas posaconazole, isavuconazole, and amphotericin B are those used for Mucorales infections (3–5). In addition, resistant isolates have been identified in many case reports (6, 7). For these reasons, the study of new fungal targets and promising antifungal molecules is an urgent need.

Nevertheless, the discovery of new antifungals is a time-consuming and highly cost-challenging task, which makes the drug repurposing approach an interesting alternative (8). The use of libraries of compounds is a promising way to optimize the identification of new molecules with antifungal activity, since it allows the fast testing of many compounds. In this context, the Medicines for Malaria Venture offers different libraries to be used against a variety of pathologies, including fungal infections. Many studies have already been published aiming to test these libraries against fungal pathogens such as *Cryptococcus*, *Candida*, *Sporothrix*, *Scedosporium*, *Mucorales*, and chromoblastomycosis agents, and they have successfully identified compounds with antifungal activity against these pathogens (9–15).

Some of these previous works tested the Pathogen Box and identified auranofin and iodoquinol as promising repurposing compounds, because both displayed antifungal activity against *Candida albicans*, *Candidozyma auris* (formerly *Candida auris*), *Sporothrix* and *Scedosporium/Lomentospora* species, and chromoblastomycosis agents (9–13). Auranofin is used for rheumatoid arthritis, and its activity is due to the inhibition of thioredoxin reductase, whereas iodoquinol chelates ferrous ions and is approved for amoebiasis (13).

Although auranofin and iodoquinol are considered promising compounds with antifungal activity, little is known about their mechanisms of action in fungal cells. For these reasons, the present study aims to evaluate the effects of both molecules against the most common species of opportunistic filamentous fungi, such as *Aspergillus fumigatus*, *Aspergillus flavus*, *Fusarium oxysporum,* and species of *Scedosporium*/*Lomentospora* and Mucorales.

## MATERIALS AND METHODS

### Strains, growth conditions, and inoculum preparation

*Aspergillus fumigatus* NCPF2109 and *Aspergillus flavus* NCPF2008 were supplied by Dr. C.K. Campbell, Mycology Reference Laboratory, Bristol Public Health Laboratory, U.K. *Fusarium oxysporum* IOC 4247 was kindly supplied by Dr. Maria Ines Sarquis from FIOCRUZ Fungi Culture Collection. *Lomentospora prolificans* FMR3569 was kindly provided by Dr. J. Guarro, Unitat de Microbiologia, Facultat de Medicina e Institut d‘Estudis Avançats, Réus, Spain. *Scedosporium boydii* CBS120157 was kindly provided by Sybren De Hoog, from the Westerdijk Fungal Biodiversity Institute, Utrecht, the Netherlands. *Rhizopus oryzae* UCP1295, isolated from the Brazilian Caatinga area, was supplied by Galba Maria de Campos-Takaki from the Culture Collection (RENNORFUN—Rede Norte Nordeste de Fungos Filamentosos) from the Catholic University of Pernambuco, Recife, Brazil. *Mucor velutinosus* H136BO and *Cunninghamella* sp. B926 were supplied by Marcio Nucci from the Mycology Laboratory of the University Hospital, Universidade Federal do Rio de Janeiro.

Fungal stocks were kept in potato dextrose agar (PDA) medium for *A. fumigatus*, *A. flavus*, *F. oxysporum*, *R. oryzae*, *M. velutinosus,* and *Cunninghamella* spp., or Sabouraud medium (0.5% yeast extract, 1% peptone, and 2% glucose monohydrate) for *S. boydii* and *L. prolificans*. To obtain conidia, cells were grown on PDA or modified Sabouraud agar plates for 7 days at room temperature. Conidia were obtained by washing the plate surface with phosphate-buffered saline (PBS, pH 7.2), and hyphal fragments and debris were removed by filtration through a Cell Strainer (Falcon, Glendale, AZ, USA). The suspension was then centrifuged, and cells were counted in Neubauer’s chamber to be used in the experiments.

### Compounds

A stock solution of each compound was prepared at 3 mM in DMSO, aliquoted, and stored at −20°C for up to 1 month. The performance of the stock solutions after storage was assessed by repeating the minimum inhibitory concentration (MIC) determination according to EUCAST methodology, and the results were consistent with those obtained using freshly prepared solutions. Voriconazole, posaconazole, and amphotericin B were used as standard antifungal agents currently employed in clinical practice and were obtained from Sigma-Aldrich (Sigma Chemical Co., USA). Auranofin and iodoquinol powder were also obtained from Sigma-Aldrich (Sigma Chemical Co., USA). The highest concentration of DMSO used in the assays was 0.68%, which decreased in each subsequent dilution. This DMSO concentration did not affect fungal growth when compared with the growth control without DMSO.

### Determination of minimal inhibitory and fungicidal concentrations against planktonic cells

The susceptibility of *A. fumigatus* NCPF2109, *A. flavus* NCPF2008, *F. oxysporum* IOC4247, *L. prolificans* FMR3569, *S. boydii* CBS120157, *R. oryzae* UCP1295, *M. velutinosus* H136BO, and *Cunninghamella* spp. B926 to auranofin and iodoquinol was determined by the broth microdilution method, according to EUCAST protocols (16, 17), with modifications described further. Voriconazole, posaconazole, and amphotericin B were also included in experiments as reference antifungals used for the treatment of infections caused by these fungi tested. Voriconazole is the reference antifungal to treat aspergillosis, fusariosis, scedosporiosis, and lomentosporiosis. Posaconazole and amphotericin B are reference antifungals to treat mucormycosis. The MIC was assessed through serial dilution of the compounds in a 96-well flat-bottom plate containing RPMI 1640 medium (Sigma-Aldrich, St. Louis, MO, USA). Auranofin, iodoquinol, posaconazole, and amphotericin B were tested at dilutions from 0.313 μM to 40 μM; however, the concentrations used for voriconazole were higher (1.25–320 μM), since the fungi tested against it show less susceptibility. Seventy percent growth inhibition was chosen as the MIC endpoint because this was the highest inhibition achieved by most of the fungi tested, and MIC_70_ was therefore selected to standardize MIC analysis across different fungal species. Standard spore suspension was added to each well at final concentrations of 2.5 × 10^5^ CFU/mL. Microplates were incubated without agitation at 37°C in 5% CO_2_ for 72 h. Although the EUCAST methodology recommends different incubation times for these fungal species, the incubation time was standardized to 72 h, since all fungi showed better growth during this period. The MIC_70_ was determined by spectrophotometry readings at 660 nm and confirmed by cell viability XTT-reduction assay assessed by readings at 492 nm (Bio-Rad, Hercules, CA, USA). Minimum fungicidal concentration (MFC) was performed after susceptibility test incubation. An aliquot of 50 μL from each well of the serial dilution was plated on PDA medium for Mucorales species, *Aspergillus* species, and *F. oxysporum*. For *Scedosporium* and *Lomentospora* species, the serial dilution was plated on Sabouraud medium. Incubation was carried out at room temperature, as all fungi tested were initially grown at room temperature to obtain spores and conidia. The plates were incubated for 48 h; however, a further 24 h incubation period can be required to get adequate growth in the control sample. The Mucorales species were observed after 48 h, and the other species were observed after 72 h. MFC values were defined as the lowest drug concentration able to completely inhibit fungal growth (18). Reference strains for internal quality control were not included in this study, which represents a methodological limitation. The antifungal activity of auranofin and iodoquinol against filamentous fungi is still poorly explored, and a wide range of fungal species was evaluated in this work. To ensure consistency and reproducibility, MIC determinations were performed using multiple independent experiments, with different replicates and freshly prepared stock solutions throughout the study. The MIC values obtained for each fungal species remained consistent across experiments.

### Kinetics of fungal growth

Conidia of the different fungi were grown in a 96-well flat-bottom plate containing RPMI 1640 medium (10^5^ cells/well). Auranofin and iodoquinol at ½ MIC concentration were added to the wells and then incubated at 37°C in 5% CO_2_. Fungi grown without auranofin or iodoquinol were used as a positive control. The growth kinetics was analyzed by optical density (OD) measured at 600 nm, every hour for 24 h of incubation using the Cytation 5 Imaging Reader (BioTek, Winooski, VT, USA) (19).

### Assays on preformed biofilms

The effect of auranofin and iodoquinol on preformed biofilm of *A. fumigatus* NCPF2109, *F. oxysporum* IOC4247, *R. oryzae* UCP1295, *M. velutinosus* H136BO, and *Cunninghamella* sp. B926 was analyzed. Briefly, a conidia suspension of each species (10^5^ cells) was added to each well in a 96-well flat-bottom plate containing RPMI 1640 medium and incubated at 37°C in 5% CO_2_ for 24 h to form biofilm. After that, the supernatant was removed, and RPMI 1640 medium was added in the absence (positive control) or presence of auranofin or iodoquinol in different concentrations (¼ MIC to 4 MIC). An additional incubation of 24 h at 37°C in 5% CO_2_ was performed to evaluate the compound’s activity. Preformed biofilms were evaluated through three parameters as previously described (20–23). Crystal violet, safranin, and XTT were used to analyze the overall biomass, extracellular matrix, and metabolic activity, respectively.

### Susceptibility to SDS, NaCl, and menadione

To evaluate the susceptibility to sodium dodecyl sulfate (SDS), NaCl, and menadione, conidia of different species (10^5^ cells/well) were grown in 96-well plates containing RPMI in the presence of auranofin or iodoquinol (½ MIC or MIC) and co-incubated with SDS, NaCl, and menadione, either in a sub-inhibitory concentration. First, the sub-inhibitory concentration for SDS, NaCl, and menadione was determined for each species by the broth microdilution method, and the concentration used for each species is demonstrated in Table 1. Positive control consisted of cells grown in the absence of auranofin or iodoquinol. After 72 h incubation at 37°C in 5% CO_2_, the cell viability was measured by using the XTT-reduction assay, and readings were captured using a spectrophotometer (Bio-Rad, Hercules, CA, USA) at 490 nm (18).

**TABLE 1:** Stressors concentrations tested against different fungal species in combination with auranofin or iodoquinol

Species	Stressors		
	SDS	NaCl	Menadione
*A. fumigatus*	45 μg/mL	2.5%	30 μM
*F. oxysporum*	45 μg/mL	1.25%	30 μM
*L. prolificans*	90 μg/mL	1%	30 μM
*S. boydii*	90 μg/mL	1%	30 μM
*R. oryzae*	45 μg/mL	1%	30 μM
*M. velutinosus*	45 μg/mL	1%	30 μM
*Cunninghamella* sp.	90 μg/mL	1%	30 μM

### Fluorescent staining to evaluate alterations in the fungal cell wall

Nile red (Sigma-Aldrich, St. Louis, MO, USA), concanavalin A (Sigma-Aldrich, St. Louis, MO, USA), and Calcofluor White (Sigma-Aldrich, St. Louis, MO, USA) were used as fluorescent probes to evaluate alterations in membrane lipids and cell wall sugars, respectively. Fungal conidia (10^5^ cells/well) were grown for 72 h at 37°C in 5% CO_2_ in the presence of auranofin or iodoquinol (¼ MIC), and untreated cells were used as a control. After a washing step, cells were stained with these fluorescent probes for 1 h at 37°C in the dark. Then, the samples were washed three times to remove residual dye and added PBS. The fluorescence intensity was measured using the SpectraMax 340 microplate reader (Molecular Devices, San Jose, CA, USA) at the following wavelengths: Nile Red at 550 nm (excitation) and 550 nm (emission), Concanavalin A at 495 nm (excitation) and 520 nm (emission), and Calcofluor White at 350 nm (excitation) and 432 nm (emission) (18, 24).

### Interaction assays of auranofin or iodoquinol with clinical antifungal drugs

Synergistic interactions were evaluated using the standard broth microdilution checkerboard method as described by Meletiadis and colleagues (25), widely used for antifungal drug-interaction studies. *Aspergillus fumigatus*, *F. oxysporum*, *L. prolificans,* and *R. oryzae* conidia (3 × 10^4^ cells/well) were grown in 96-well plates containing supplemented RPMI in the presence of the following selected compounds: Auranofin and iodoquinol, combined with voriconazole (0.31–40 µM), posaconazole (0.31–40 µM), or amphotericin B (0.031–5 µM). After incubation for 72 h at 37°C in 5% CO_2_, MIC was evaluated at 660 nm, and cell viability was assessed via the XTT reduction assay at 490 nm. An inhibition of at least 70% was defined as the cut-off for MIC. Interactions were determined by the fractional inhibitory concentration index (FICI), which was calculated using the following formula: (MIC combined/MIC drug A alone) + (MIC combined/MIC drug B alone). The results were classified as follows: synergistic effect, FICI of ≤0.5; additive effect, FICI >5-1; no effect, FICI of >1-4.0; antagonistic effect, FICI of >4.0 (25). The Bliss independence model calculation was performed according to Meletiadis and colleagues and Zhao and colleagues (26, 27). The following formula was used to assess drug interaction: *E*_exp_ = *E*a + *E*b – *E*a × *E*b, in which *E*_exp_ is the expected efficacy of the drug combination, *E*a is the efficacy of drug A (auranofin and iodoquinol), and *E*b is the efficacy of drug B (voriconazole, posaconazole, or amphotericin B). The results were classified as follows: synergistic effect when *E*_obs_ > *E*_exp_; indifference when *E*_obs_ = *E*_exp_; antagonistic effect when *E*_obs_ < *E*_exp_.

### Scanning electron microscopy 

*Aspergillus fumigatus* NCPF2109 and *F. oxysporum* IOC4247 were grown in RPMI in the presence of ¼ and ½ MIC_70_ of auranofin and iodoquinol with orbital agitation (150 rpm) for 72 h. Positive control consisted of cells grown in the absence of these compounds. Mycelium samples were gently collected and processed via the following steps: (i) fixation in 2.5% glutaraldehyde and 4% formaldehyde in 0.1 M cacodylate buffer and 10 mM CaCl_2_ for at least 24 h at 4°C, (ii) deposition onto round coverslips previously coated with poly-L-lysine for 20 min at room temperature; (iii) three washes in 0.1 M cacodylate buffer, followed by post-fixation in 1% osmium tetroxide with 0.8% potassium ferrocyanide in 0.1 M cacodylate buffer for 1 h; (iv) three washes in 0.1 M cacodylate buffer, followed by dehydration through a graded ethanol series (30%–100%); (v) critical point drying with CO₂; (vi) mounting of the coverslips onto aluminum stubs using conductive carbon tape; and (vii) sputter-coating with gold. Images were acquired using a Thermo Quattro S FEG scanning electron microscope (SEM; Thermo Fisher Scientific Inc., Waltham, Massachusetts, USA). The acquired images were processed using Adobe Photoshop software (version 24.6.0, San Jose, CA, USA).

### Cytotoxicity assay

Cytotoxicity assays for auranofin and iodoquinol were conducted using the human alveolar basal epithelial adenocarcinoma cell line (A549). Cells were cultured in Dulbecco’s Modified Eagle’s Medium (DMEM; Sigma-Aldrich, MO, USA) supplemented with 10% fetal bovine serum and seeded into 96-well plates to form confluent monolayers after 24 h of incubation at 37°C in a humidified atmosphere containing 5% CO₂. Cell monolayers were then exposed to serial dilutions of each compound (0.78–50 µM) for 2 h or 24 h under the same incubation conditions. Cell viability was subsequently assessed using the Neutral Red (NR) uptake assay, and absorbance was measured at 595 nm using a SpectraMax i3x microplate reader (Molecular Devices, San José, CA, USA).

### Statistical analyses

All experiments were performed in triplicate, in three independent experimental sets. Statistical analyses were performed using GraphPad Prism version 5.00 for Windows (GraphPad Software, San Diego, CA, USA). One-way ANOVA was used for multiple comparisons using Turkey’s test (Turkey post-test), and a t-test was used for two-group comparisons. The 90% or 95% confidence interval was determined in all experiments.

## RESULTS

### Auranofin and iodoquinol are able to inhibit fungi from the WHO list of priority fungal pathogens

Auranofin and iodoquinol are promising repurposing compounds with previously demonstrated antifungal activity (9, 12, 13, 28, 29). Based on the World Health Organization (WHO) fungal priority pathogens list published in 2022, eight species from different priority groups were selected: *Aspergillus fumigatus* (critical); *Fusarium oxysporum*, Mucorales species (*R. oryzae*, *M. velutinosus,* and *Cunninghamella* sp.), and *Scedosporium boydii* (high); and *Lomentospora prolificans* (medium). In addition, *Aspergillus flavus* was included due to its clinical relevance as a major cause of aspergillosis and fungal rhinosinusitis (30–32). Notably, *S. boydii* and *L. prolificans* had been previously tested against auranofin and iodoquinol in an earlier study (13); however, the MFCs for these fungi were not tested before.

The MICs for auranofin and iodoquinol across eight different species are demonstrated in Table 2. Voriconazole, posaconazole, and amphotericin B have also been tested, as they are the first choice for treating these infections in clinical settings. The MIC was able to inhibit 70% of the fungal growth for auranofin, which ranged from 5 µM to >40 µM. The MIC_70_ for *A. fumigatus*, *L. prolificans,* and *S. boydii* was 5 µM, for *A. flavus* and *F. oxysporum* was 10 µM, for *R. oryzae* and *Cunninghamella* sp. was 20 µM, and *M. velutinosus* showed the MIC_70_ > 40 µM because the auranofin was not able to inhibit the growth up to 40 µM, the highest concentration tested in this study. Auranofin demonstrated fungicidal activity against both *Aspergillus* species, *L. prolificans*, *S. boydii*, and *R. oryzae*, with MFCs ranging from 10 to 40 µM. However, auranofin exhibited only fungistatic activity against *F. oxysporum* and *Cunninghamella* sp. as MFCs were >40 µM. The MIC_70_ values for iodoquinol ranged from 0.63 to >40 µM. The most susceptible species to iodoquinol was *S. boydii* (MIC_70_ 0.63 µM), followed by *A. flavus* and *L. prolificans* (MIC_70_ 1.25 µM). *A. fumigatus* and *F. oxysporum* showed MIC_70_ values of 2.5 and 5 µM, respectively. Among the Mucorales species, *R. oryzae* had an MIC_70_ of 20 µM, while *M. velutinosus* and *Cunninghamella* sp. were the most resistant, with MIC_70_ values > 40 µM. Iodoquinol exhibited a fungistatic profile against most of the tested species, as they continued to grow at the highest concentration tested in this study. Iodoquinol showed no activity against *M. velutinosus* or *Cunninghamella* sp. at the highest concentration tested. The species most susceptible to voriconazole were *A. fumigatus*, *A. flavus*, and *S. boydii*, each with an MIC_70_ of 1.25 µM. In contrast, *F. oxysporum* and *L. prolificans* were the most resistant, with MIC_70_ values of 80 and 160 µM, respectively. Voriconazole exhibited fungicidal activity against both *Aspergillus* species and *S. boydii*, with MFCs ranging from 2.5 to 10 µM. However, it was not able to kill *F. oxysporum* and *L. prolificans*, with MFCs > 80 µM and > 320 µM, respectively. Among the Mucorales species, *R. oryzae* was the most susceptible to both posaconazole (MIC_70_ 5 µM) and amphotericin B (MIC_70_ 0.31–0.63 µM), followed by *Cunninghamella* sp. (MIC_70_ 10 µM for posaconazole and 5–10 µM for amphotericin B). *Mucor velutinosus* was the most resistant species to both drugs, with MIC values > 40 µM. Neither posaconazole nor amphotericin B demonstrated fungicidal activity against the three Mucorales species tested, as MFCs were > 40 µM.

**TABLE 2:** Minimal inhibitory and fungicidal concentration of auranofin, iodoquinol, voriconazole, posaconazole, and amphotericin B against different fungal species^*a*^

Species	Auranofin		Iodoquinol		Voriconazole			
	MIC_70_	MFC	MIC_70_	MFC	MIC_70_		MFC	
*Aspergillus fumigatus*	5	10	2.5	> 40	1.25		2.5	
*Aspergillus flavus*	10	–	1.25	> 40	1.25		–	
*Fusarium oxysporum*	10	> 40	5	> 40	80		> 80	
*Lomentospora prolificans*	5	40	1.25	> 40	160		> 320	
*Scedosporium boydii*	5	10	0.625	> 40	1.25		10	

Species	Auranofin		Iodoquinol		Posaconazole		Amphotericin B	
	MIC_70_	MFC	MIC_70_	MFC	MIC_70_	MFC	MIC_70_	MFC
*Rhizopus oryzae*	20	40	20	> 40	5	> 40	0.31-0.62	> 40
*Mucor velutinosus*	> 40	> 40	> 40	> 40	> 40	> 40	> 40	> 40
*Cunninghamella* sp.	20	> 40	> 40	> 40	10	> 40	5-10	> 40

^*a*^ MIC_70_: minimum inhibitory concentration able to inhibit 70% of the fungal growth. MFC: minimum fungicidal concentration. –, not determined.

### Fungal growth kinetics under the influence of auranofin and iodoquinol

**Fig 1:**
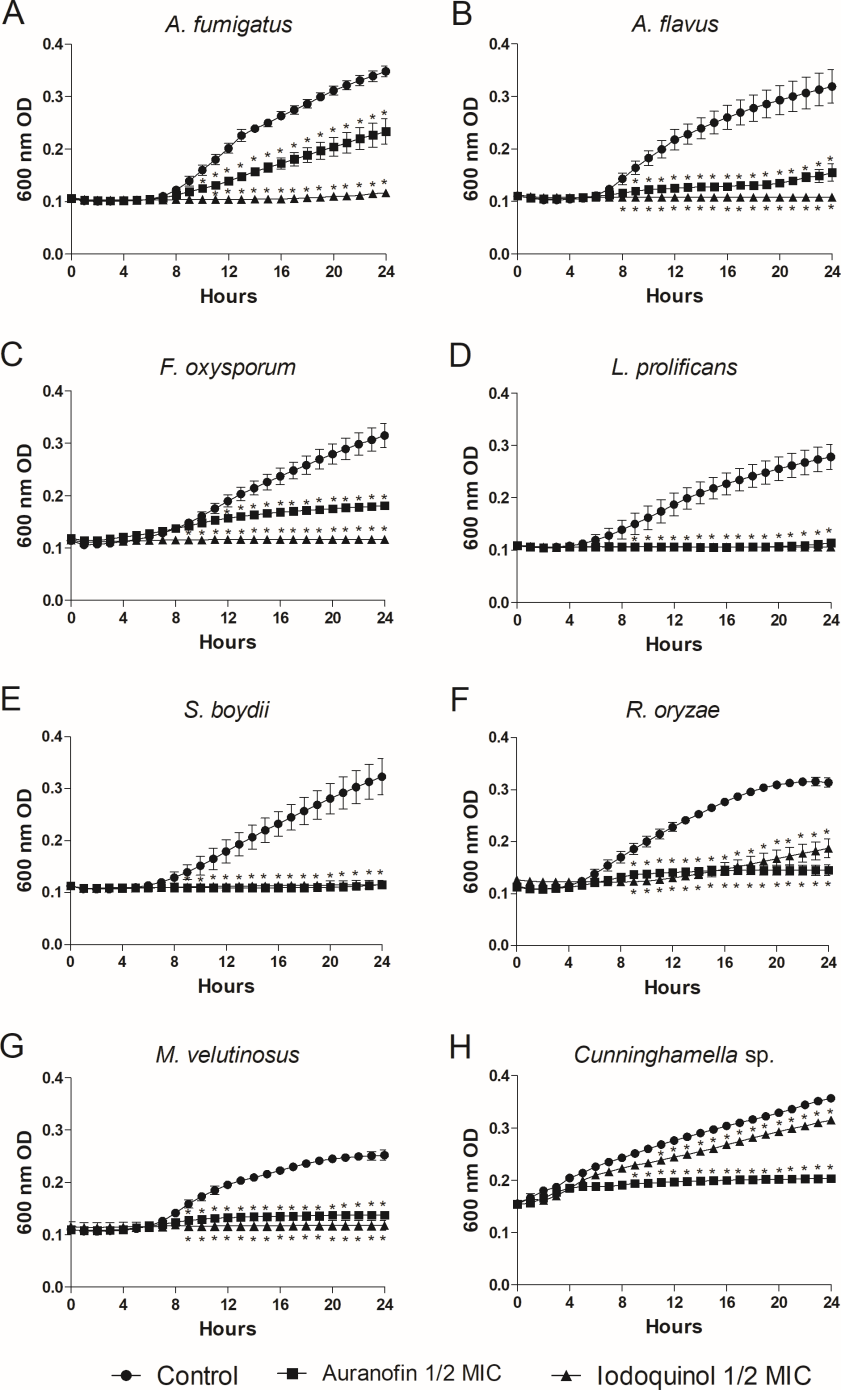
Fungal growth kinetics under the influence of auranofin and iodoquinol. *Aspergillus fumigatus* (**A**), *Aspergillus flavus* (**B**), *Fusarium oxysporum* (**C**), *Lomentospora prolificans* (**D**), *Scedosporium boydii* (**E**), *Rhizopus oryzae* (**F**), *Mucor velutinosus* (**G**), and *Cunninghamella* sp. (**H**). Cells were incubated in the absence (Control) or the presence of ½ MIC of auranofin or iodoquinol at 37°C for up to 24 h; the OD was measured every 1 h. *P < 0.001.

Growth kinetics in the presence of ½ MIC of auranofin or iodoquinol was performed to observe the inhibitory effect throughout the first 24 h of incubation, at 37°C and 5% of CO_2_ (Fig. 1). All eight species were analyzed. In untreated samples (control), fungal growth was observed from 6 to 8 h, except for *Cunninghamella* sp. that grew after only 2 h of incubation (Fig. 1H). Auranofin partially inhibited *A. fumigatus* growth significantly after 10 h of incubation (Fig. 1A). However, auranofin almost completely inhibited the *A. flavus* growth after 8 h of incubation and kept inhibition up to 20 h, when a slight increase in growth began (Fig. 1B). Auranofin completely inhibited the growth of *L. prolificans*, *S. boydii,* and *M. velutinosus* up to 24 h (Fig. 1D, E and G). *F. oxysporum* was partially inhibited by auranofin with a significant difference after 12 h of incubation (Fig. 1C). Auranofin inhibited *R. oryzae* growth from 7 to 24 h of incubation (Fig. 1F). Although the growth of *Cunninghamella* sp. started earlier than the other fungi, the inhibition by auranofin began after 10 h of incubation and maintained a low percentage of inhibition up to 24 h of incubation (Fig. 1H). Iodoquinol was able to almost completely inhibit the species tested from 7 or 8 h to 24 h, except for *R. oryzae,* which started to grow after 20 h of incubation (Fig. 1).

### Influence of auranofin and iodoquinol on fungal biofilms

**Fig 2:**
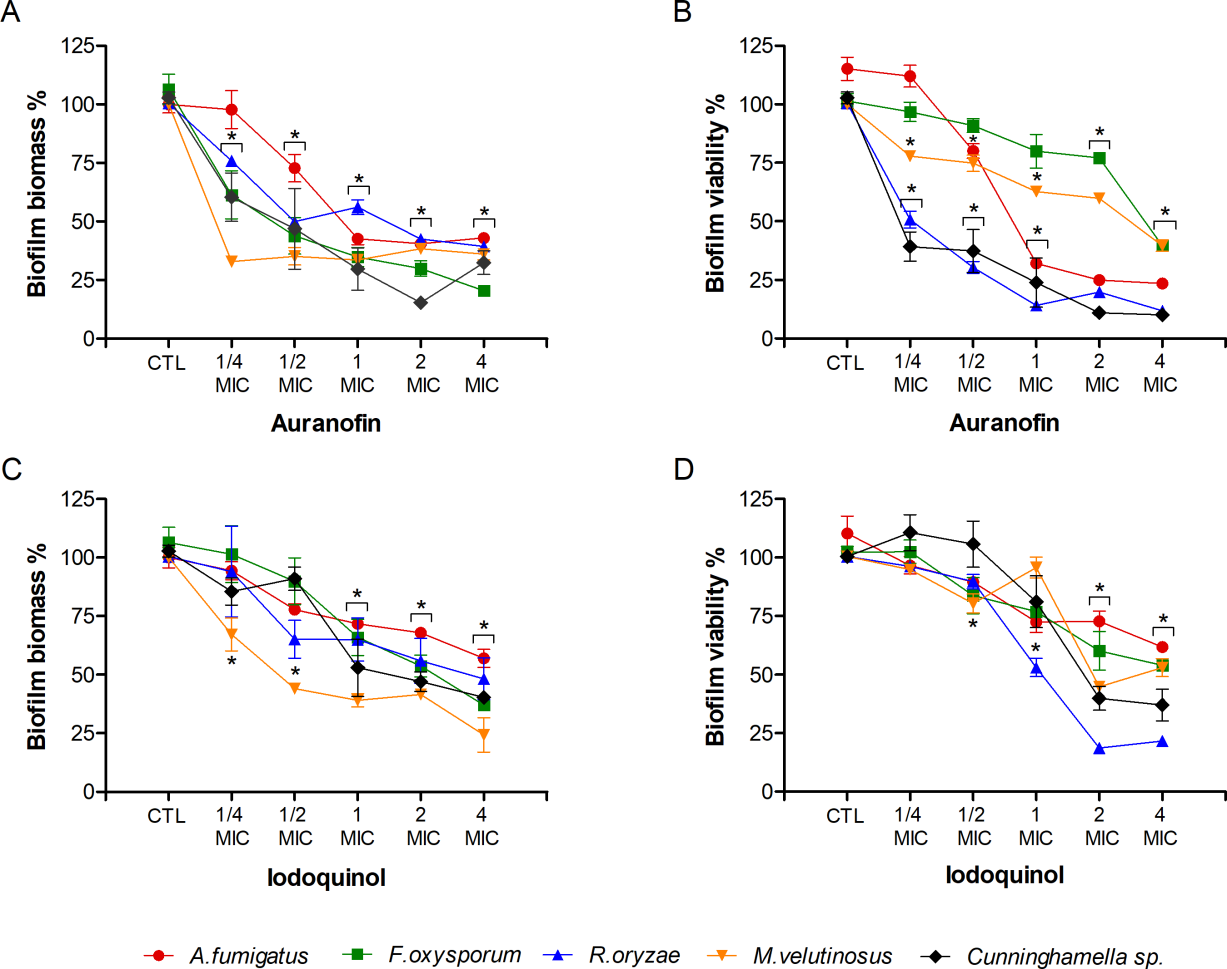
Influence of auranofin and iodoquinol on fungal biofilms. Fungal biofilm was first formed in RPMI 1640 medium on polystyrene surface for 24 h, and then it was treated with different concentrations of auranofin and iodoquinol (¼–4 MIC) for another 24 h incubation. Intact fungal biofilms were considered controls (CTL, 100% biofilm), and their degradation due to treatment was compared to the control. Fungal biomass (**A and C**) and viability (**B and D**) were measured using violet crystal and XTT reduction assay, respectively. *P < 0.05, compared to control (absence of drug) for each species.

The ability to form biofilms has been demonstrated for almost all pathogenic fungi and is associated with antifungal resistance since the biofilm structure reduces the efficacy of drugs used to treat fungal infections (33–35). Therefore, the effect of auranofin and iodoquinol on preformed biofilms was analyzed. Preformed biofilm analysis for *L. prolificans*, *S. aurantiacum,* and *S. boydii* treated with auranofin and iodoquinol has been demonstrated in a previous work (13), and its percentage of inhibition at 4 MIC is shown in Table 3. For biofilm analysis, one species from each genus was chosen for this analysis based on its clinical relevance, and this selection parameter was also considered for the following tests. Auranofin reduced the biomass of all fungal biofilms tested, from the ¼ MIC concentration, with the exception of *A. fumigatus* biofilm, which was reduced from the ½ MIC (Fig. 2A). At the highest concentration tested (4 MIC), auranofin reduced more than 50% of the biomass and viability of the biofilms tested (Fig. 2A and B). The *F. oxysporum* and *M. velutinosus* biofilm showed less susceptibility to auranofin, since the reduction of biofilm viability was 60% at 4 MIC concentration (Table 3). Iodoquinol was able to reduce the biomass of all fungal biofilm tested at MIC concentration (Fig. 2C). Iodoquinol was able to inhibit more than 50% of all fungal biofilms tested at the 4 MIC concentration, except for the *A. fumigatus* preformed biofilm, which reached a maximum of 40% inhibition at the 4 MIC concentration (Table 3). Analyzing the viability of the biofilm treated with iodoquinol, it was possible to observe that the preformed biofilms of *R. oryzae* and *Cunninghamella* sp. were more susceptible at the 4 MIC concentration, presenting a reduction of 80% and 60%, respectively (Fig. 2D). The preformed biofilm from *L. prolificans*, *S. aurantiacum*, and *S. boydii* was also susceptible to the auranofin and iodoquinol treatment, which was shown in the previous work (13). Auranofin was able to decrease 90% of the *L. prolificans* preformed biofilm and 80% of the *S. aurantiacum* and *S. boydii* preformed biofilm at 4 MIC; however, iodoquinol decreased up to 70% of the *L. prolificans* and *S. aurantiacum* preformed biofilm and 50% of the *S. boydii* preformed biofilm (Table 3).

**TABLE 3:** Viability inhibition of preformed biofilms of different fungal species^*a*^

	Preformed biofilm				
Species	Auranofin		Iodoquinol		Reference
	4 MIC_70_	Inhibition	4 MIC_70_	Inhibition	
*A. fumigatus*	40 µM	80%	5 µM	40%	This work
*F. oxysporum*	80 µM	60%	10 µM	50%	This work
*L. prolificans*	20 µM	90%	2.5 µM	70%	Rollin-Pinheiro 2021
*S. aurantiacum*	20 µM	80%	20 µM	70%	Rollin-Pinheiro 2021
*S. boydii*	20 µM	80%	2.5 µM	50%	Rollin-Pinheiro 2021
*R. oryzae*	80 µM	90%	80 µM	80%	This work
*M. velutinosus*	160 µM	60%	160 µM	50%	This work
*Cunninghamella* sp.	80 µM	90%	160 µM	60%	This work

^*a*^ 4 MIC70: fourfold the minimum inhibitory concentration.

### Susceptibility to stressors in the presence of auranofin and iodoquinol

**Fig 3:**
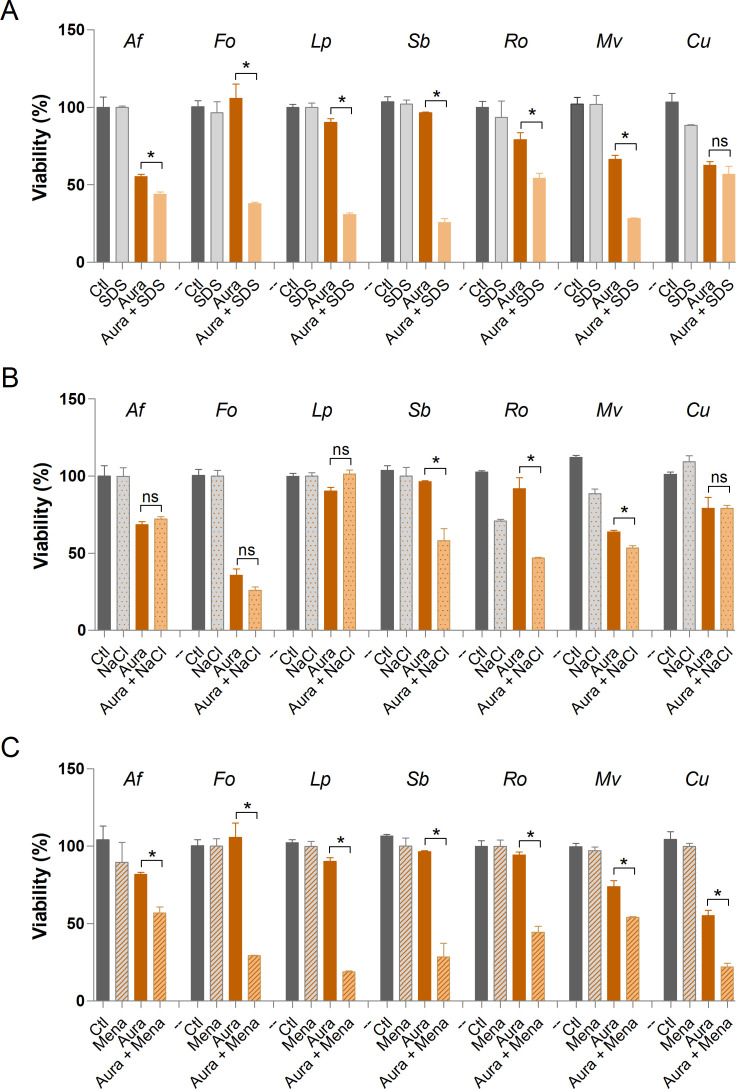
Susceptibility of different fungal species to stressors in the presence of auranofin. SDS was used as a membrane stressor (**A**). NaCl was used as an osmotic stressor (**B**). Menadione used as an oxidative stressor (**C**). The control represents fungal viability in the absence of stressors and auranofin. Af: *A. fumigatus*; Fo: *F. oxysporum*; Lp: *L. prolificans*; Sb: *S. boydii*; Ro: *R. oryzae*; Cu: *Cunninghamella* spp.; Mv: *M. velutinosus*; Ctl: Control; Aura: Auranofin; SDS: Sodium dodecyl sulfate; Mena: Menadione. * *P* < 0.05, ns: not significant. Experiments were performed in duplicate in three independent experimental sets.

**Fig 4:**
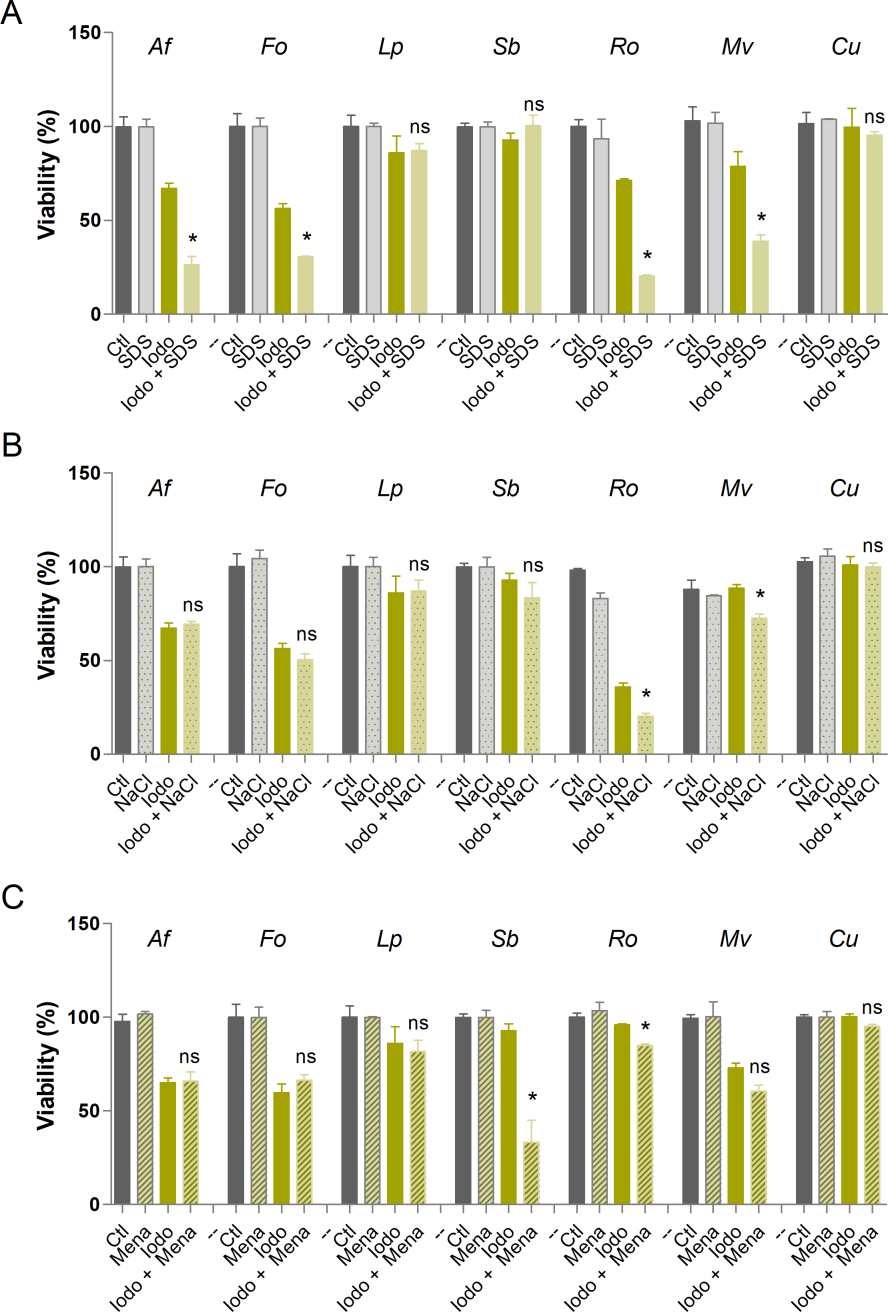
Susceptibility of different fungal species to stressors in the presence of iodoquinol. SDS was used as a membrane stressor (**A**). NaCl was used as an osmotic stressor (**B**). Menadione was used as an oxidative stressor (**C**). The control represents fungal viability in the absence of stressors and iodoquinol. Af: *A. fumigatus*; Fo: *F. oxysporum*; Lp: *L. prolificans*; Sb: *S. boydii*; Ro: *R. oryzae*; Cu: *Cunninghamella* spp.; Mv: *M. velutinosus*; Ctl: Control; Iodo: Iodoquinol; SDS: Sodium dodecyl sulfate; Mena: Menadione. *P < 0.05, ns: not significant. Experiments were performed in duplicate in three independent experimental sets.

Although the primary mechanisms of action of auranofin and iodoquinol are known, these compounds may have multiple molecular targets across different microorganisms (36, 37). To investigate their effects on different fungal species, fungal susceptibility to auranofin and iodoquinol under conditions of cellular stress was assessed. Three stress-inducing agents were employed: SDS, an anionic detergent that disrupts membrane integrity; NaCl, which induces osmotic stress; and menadione, a compound that generates oxidative stress. The species *A. fumigatus*, *F. oxysporum*, *L. prolificans*, *S. boydii*, *R. oryzae*, *M. velutinosus,* and *Cunninghamella* sp. were tested in the absence of any compound or stressor as a growth control, and in the presence of sub-inhibitory concentrations of each stressor, which did not decrease the viability compared to the control. Auranofin or iodoquinol was tested alone and in the presence of each stressor at ½ MIC concentration (Fig. 3 and 4). Auranofin was able to increase the susceptibility of all fungi tested in the presence of SDS, with the exception of *Cunninghamella* sp., suggesting its effect on membrane integrity (Fig. 3A). In the presence of NaCl, auranofin increased the susceptibility of only *S. boydii*, *R. oryzae,* and *M. velutinosus* species (Fig. 3B). Auranofin increased the susceptibility of all fungi tested in the presence of menadione (Fig. 3C), which is consistent with its main mechanism of action that involves the inhibition of cellular redox proteins (14). Iodoquinol was able to increase only the susceptibility of *A. fumigatus*, *F. oxysporum*, *R. oryzae,* and *M. velutinosus* in the presence of SDS (Fig. 4A). Only *R. oryzae* and *M. velutinosus* exhibited decreased viability in the presence of both iodoquinol and NaCl (Fig. 4B). In the presence of menadione, iodoquinol increased the susceptibility of only *S. boydii* and *R. oryzae* (Fig. 4C).

### Evaluation of cellular alterations induced by auranofin and iodoquinol

**Fig 5:**
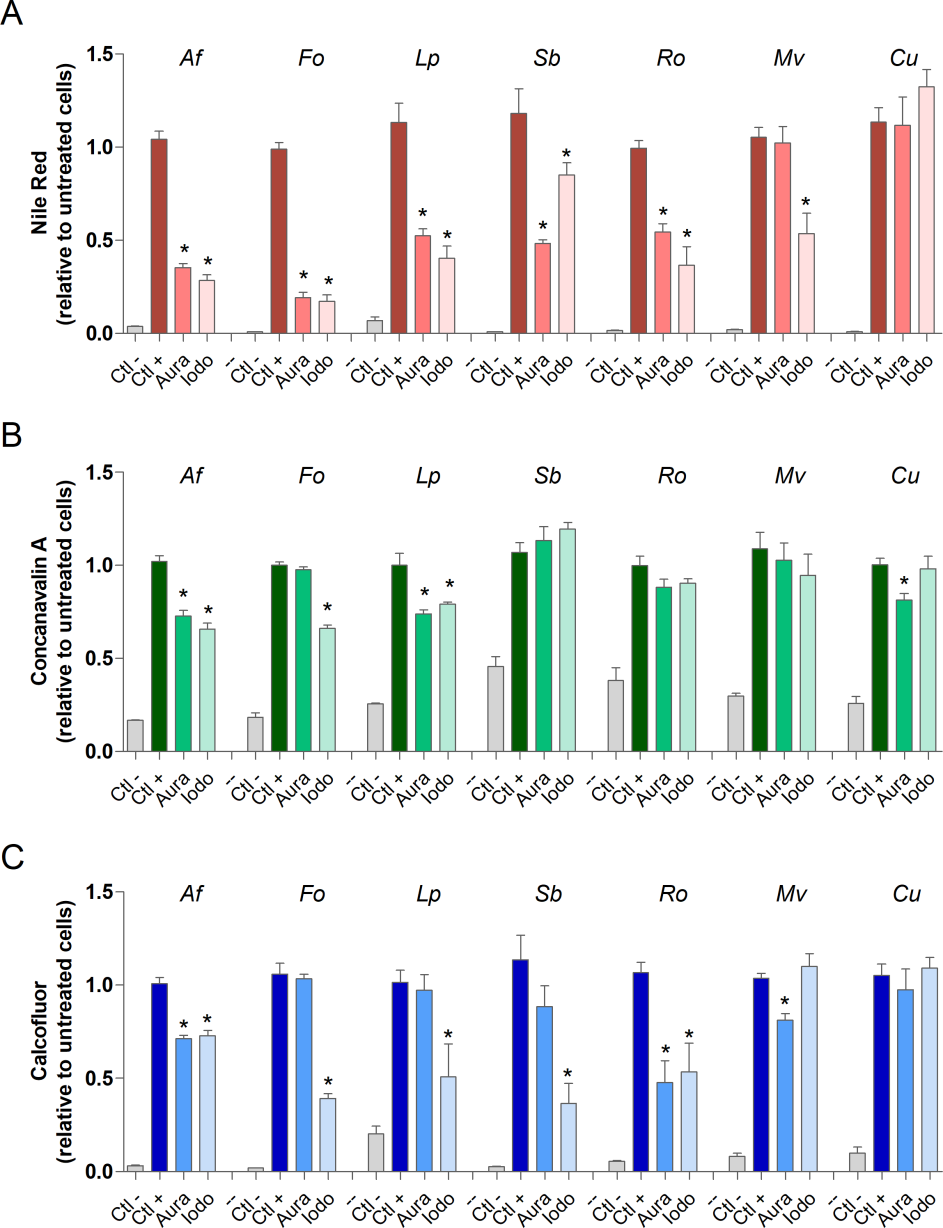
Cellular alterations induced by auranofin and iodoquinol were analyzed by fluorescent staining. Cells were grown in the presence of ¼ MIC_70_ for 72 H at 37°C and 5% CO_2_. Neutral lipids were quantified using Nile Red stain (**A**). Concanavalin A was used to evaluate mannose (**B**). Chitin content was analyzed using calcofluor white (**C**). Ctl (−), a negative control that represents untreated cells in the absence of fluorescent stain. Ctl (+), a positive control that represents untreated cells stained with fluorescent stain. Aura: auranofin; Iodo: Iodoquinol *P < 0.05; ns: not significant.

For the purpose of identifying the alterations in different fungal cells caused by auranofin and iodoquinol, each species was grown in the presence of ¼ MIC of these compounds for 72 h. Then, fluorescent staining procedures were used to measure cellular alterations. Untreated cells were used as controls. The contents of neutral lipids, mannose residues, and chitin were analyzed using Nile Red, Concanavalin A, and Calcofluor White, respectively. Both auranofin and iodoquinol drastically decreased the neutral lipid content in *A. fumigatus*, *F. oxysporum*, *L. prolificans*, *S. boydii*, and *R. oryzae*. However, neither compound affected *Cunninghamella* sp., while a reduction in neutral lipids in *M. velutinosus* was observed only with iodoquinol treatment (Fig. 5A). Auranofin and iodoquinol significantly reduced the mannose content in *A. fumigatus* and *L. prolificans*; however, neither compound affected *S. boydii*, *R. oryzae,* and *M. velutinosus*, while a reduction in mannose content in *F. oxysporum* was observed only with iodoquinol treatment and in *Cunninghamella* sp. was observed only with auranofin treatment (Fig. 5B). With respect to chitin content analysis, both auranofin and iodoquinol significantly reduced chitin levels in *A. fumigatus* and *R. oryzae*. In *F. oxysporum*, *L. prolificans*, and *S. boydii*, only iodoquinol was able to reduce chitin content, whereas a slight reduction in chitin levels was observed in *M. velutinosus* in the presence of auranofin. Neither compound altered chitin content in *Cunninghamella* sp. (Fig. 5C).

### Analysis of drug interactions between auranofin and iodoquinol with conventional antifungals

Due to auranofin and iodoquinol exhibiting antifungal activity against different fungal species and inducing alterations in their cells, we sought to investigate whether these compounds would interact with antifungal agents currently employed in clinical settings. The interaction of auranofin and iodoquinol with antifungal agents was evaluated using both the FICI and Bliss methods and tested against *A. fumigatus*, *F. oxysporum*, *L. prolificans,* and *R. oryzae*, which were chosen based on their clinical relevance (Tables 4 and 5). According to the FICI analysis, *A. fumigatus* showed a synergistic effect with iodoquinol and voriconazole, while auranofin combined with voriconazole presented an additive effect. For *F. oxysporum* and *L. prolificans*, both auranofin and iodoquinol in combination with voriconazole also showed an additive effect. Regarding *R. oryzae*, no interaction was observed with either auranofin or iodoquinol combined with posaconazole, but an additive effect was seen in combination with amphotericin B (Table 4). In the Bliss analysis, *A. fumigatus*, *F. oxysporum*, and *L. prolificans* all exhibited synergistic interactions with voriconazole when combined with auranofin or iodoquinol. For *R. oryzae*, an antagonistic interaction was observed with posaconazole and both auranofin and iodoquinol, while a synergistic effect was seen with amphotericin B (Table 5).

**TABLE 4:** Antifungal activity of auranofin, iodoquinol, voriconazole, posaconazole, and amphotericin B—alone and in combination according to the FICI—against *A. fumigatus*, *F. oxysporum*, *L. prolificans,* and *R. oryzae*^*a*^^,^^*b*^

Species	MIC alone (µM)		MIC combined (µM)		FICI
*A. fumigatus*	Auranofin	5	Aura/Vori	2.5/0.625	1.0 (additive)
	Iodoquinol	2.5	Iodo/Vori	0.15/0.31	0.31 (synergistic)
	Voriconazole	1.25			
*F. oxysporum*	Auranofin	10	Aura /Vori	5/40	1.0 (additive)
	Iodoquinol	5	Iodo/Vori	2.5/40	1.0 (additive)
	Voriconazole	80			
*L. prolificans*	Auranofin	5	Aura /Vori	2.5/40	0.75 (additive)
	Iodoquinol	1.25	Iodo/Vori	0.3/80	0.75 (additive)
	Voriconazole	160			
*R. oryzae*	Auranofin	20	Aura/Posa	10/5	2.0 (no effect)
	Iodoquinol	20	Iodo/Posa	20/5	2.0 (no effect)
	Posaconazole	5	Aura/AmphB	5/0.16	0.75 (additive)
	Amphotericin B	0.32	Iodo/AmphB	2.5/0.08	0.375 (additive)

^*a*^ MIC: minimum inhibitory concentration; Aura: auranofin; Iodo: iodoquinol; Vori: voriconazole; Posa: posaconazole; AmphB: amphotericin B.

^*b*^ MIC_70_ values were used to analyze the interaction between auranofin or iodoquinol with voriconazole, posaconazole, or amphotericin B.

**TABLE 5:** Antifungal activity of auranofin, iodoquinol, voriconazole, posaconazole, and amphotericin B—alone and in combination according to the bliss independence method—against *A. fumigatus*, *F. oxysporum*, *L. prolificans,* and *R. oryzae*^*a*^

		Efficacy of drugs alone (% of inhibition)		Efficacy of combined drugs					
				Auranofin			Iodoquinol		
		MIC	½ MIC	*E*_obs_	*E*_exp_	Δ*E*, % (interaction)	*E*_obs_	*E*_exp_	ΔE, % (interaction)
*A. fumigatus*	Vori	83.3	60.4	86.3	72.3	14 (S)	89.3	52.9	36.4 (S)
	Aura	89.6	53.2	ND	ND	ND	ND	ND	ND
	Iodo	86.1	24.8	ND	ND	ND	ND	ND	ND
*F. oxysporum*	Vori	71.7	43.4	80	56.4	23.6 (S)	74.5	73.8	0.7 (S)
	Aura	84.6	55.6	ND	ND	ND	ND	ND	ND
	Iodo	82.5	54.8	ND	ND	ND	ND	ND	ND
*L. prolificans*	Vori	72.9	30.6	76	63.6	12.4 (S)	80.7	72	8.7 (S)
	Aura	85.2	34.8	ND	ND	ND	ND	ND	ND
	Iodo	80.4	22.3	ND	ND	ND	ND	ND	ND
*R. oryzae*	Posa	70.0	40.5	39	46.1	−7.1 (A)	33.1	40.2	−7.1 (A)
	Ampho B	89.3	26.4	92.7	54.9	37.8 (S)	90.0	59.9	30.1 (S)
	Aura	89.8	54.5	ND	ND	ND	ND	ND	ND
	Iodo	88.3	41.5	ND	ND	ND	ND	ND	ND

^*a*^ MIC: Minimum inhibitory concentration. *E*_obs_: efficacy observed in the analysis. *E*_exp_: efficacy expected according to Bliss calculation. *ΔE*: difference between *E*_obs_ and *E*_exp_. ND, not determined S: synergistic interaction. A: antagonist interaction.

### Cellular morphology alterations induced by auranofin and iodoquinol

**Fig 6:**
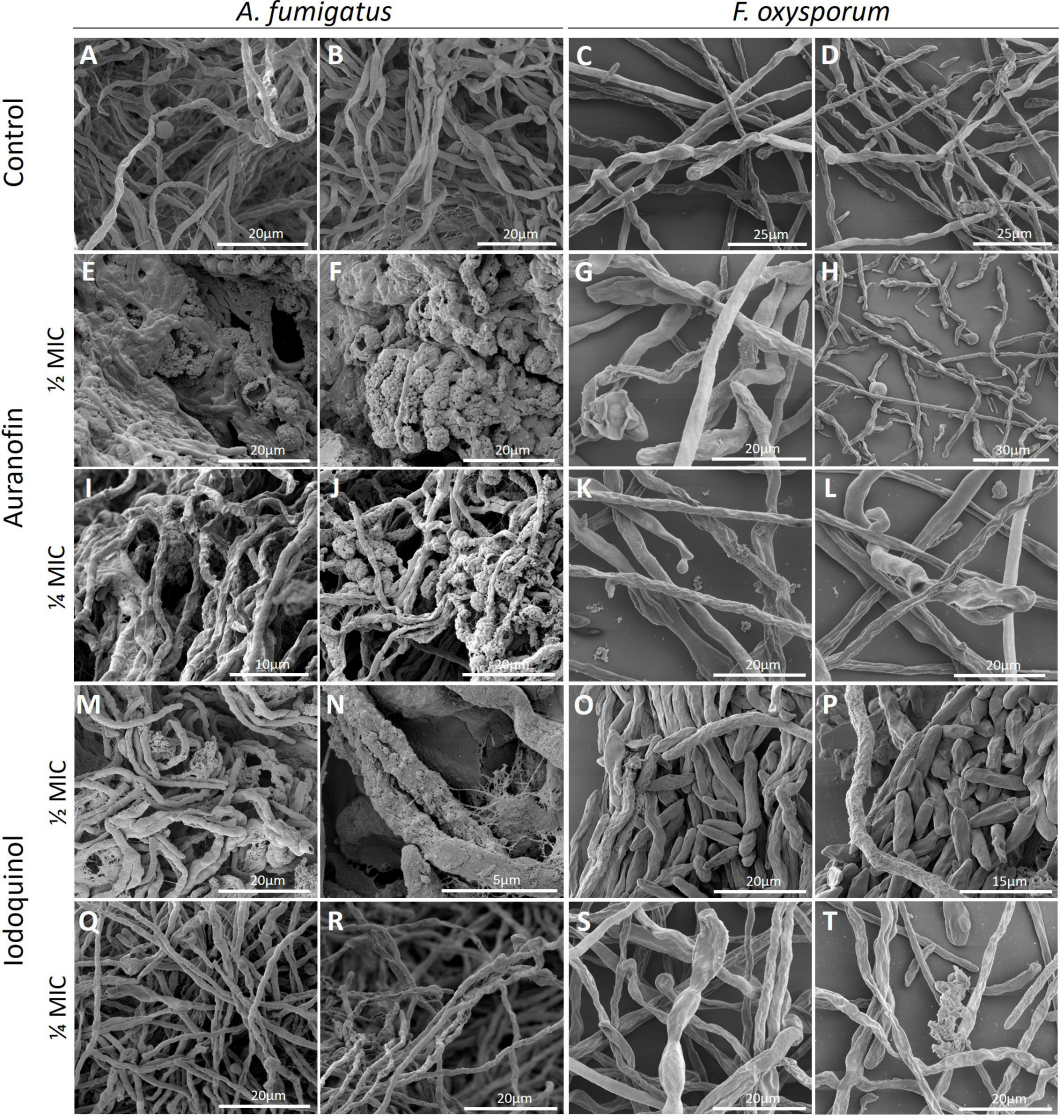
Scanning electron microscopy of *A. fumigatus* and *F. oxysporum* treated with ½ or ¼ MIC of auranofin or iodoquinol. The control condition represents both fungi grown in the absence of the compounds. Some regions show accumulation of extracellular substances.

Since auranofin and iodoquinol caused cellular alterations in fungal cells, the morphology of *A. fumigatus* and *F. oxysporum* treated with these drugs was conducted by Scanning Electron Microscopy (SEM). These species were selected since they are the most common filamentous fungi implicated in human infection and experimental antifungal susceptibility testing (2, 38). Both species were grown for 72 h in the presence of sub-inhibitory concentrations of auranofin and iodoquinol (¼ and ½ MIC_70_) (Fig. 6). In the absence of any drug, the *A. fumigatus* mycelium was formed, and it was possible to observe a small amount of extracellular polymeric substance (Fig. 6A and B). In the presence of ½ MIC of auranofin, *A. fumigatus* secreted an extensive amount of extracellular substance, with the hyphae immersed in this substance, becoming them thicker and more united, similar to a biofilm (Fig. 6E). In addition, it is possible to observe the formation of a dense extracellular matrix organized into lobular clusters with a raspberry-like appearance (Fig. 6F). Similar morphological alterations were also observed at the lowest auranofin concentration tested against *A. fumigatus* (¼ MIC), although the extracellular polymeric matrix appeared less abundant (Fig. 6I and J). *Aspergillus fumigatus* exposed to ½ MIC of iodoquinol produced an abundant extracellular matrix, which formed a reticulated layer around the hyphae, resulting in a thicker appearance (Fig. 6M and N). The concentration of ¼ MIC of iodoquinol, *A. fumigatus,* secreted a small amount of extracellular matrix, similar to the control (Fig. 6Q and R). *Fusarium oxysporum* control grew forming either smooth or rough hyphae, and some conidia can be observed (Fig. 6C and D). Exposure to ½ MIC of auranofin reduced *F. oxysporum* growth and led to the formation of numerous short hyphae and ungerminated conidia (Fig. 6G), as well as increased extracellular material on some hyphal surfaces (Fig. 6H). Incubation with ¼ MIC of auranofin resulted in hyphae displaying little extracellular material and few short hyphae (Fig. 6K and L). In the presence of ½ MIC of iodoquinol, most *F. oxysporum* conidia did not germinate, and only a few of the formed hyphae exhibited extracellular material on their surfaces (Fig. 6O and P). Incubation of *F. oxysporum* with ¼ MIC of iodoquinol resulted in hyphae displaying a small amount of extracellular material (Fig. 6S and T).

### Cytotoxicity of auranofin and iodoquinol in A549 cells

**Fig 7:**
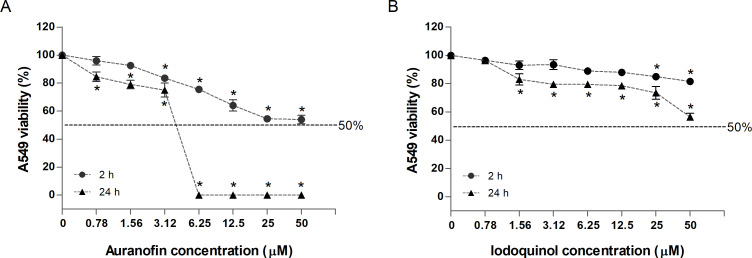
Cytotoxicity of auranofin and iodoquinol in A549 cells. Human lung epithelial A549 cells were exposed to increasing concentrations of auranofin (**A**) or iodoquinol (**B**) for 2 h and 24 h, and cell viability was determined using the neutral red uptake assay. Auranofin showed limited cytotoxicity at lower concentrations, with reduced cell viability at higher doses and prolonged exposure, whereas iodoquinol maintained relatively high cell viability even at higher concentrations. Data are expressed as a percentage of viable cells relative to untreated controls. *P < 0.05.

Cytotoxicity of auranofin and iodoquinol was evaluated in human lung epithelial A549 cells using time- and concentration-dependent viability analyzes (Fig. 7). For auranofin, treatment at 3.12 µM resulted in 83% cell viability after 2 h of incubation and 77% viability after 24 h (Fig. 7A). Increasing the concentration to 6.25 µM reduced cell viability to 75% after 2 h, while prolonged exposure to 6.25 µM for 24 h led to complete loss of viable cells, indicating a pronounced time- and dose-dependent cytotoxic effect. Notably, after 2 h of incubation, auranofin did not reduce cell viability below 50% even at the highest concentration tested (50 µM), indicating limited acute cytotoxicity at short exposure times. For iodoquinol, A549 cells maintained approximately 85% viability after 2 h of incubation at concentrations up to 25 µM, and 73% viability after 24 h at the same concentration (Fig. 7B). Importantly, iodoquinol did not reduce cell viability below 50% at any concentration tested, including the highest concentration (50 µM), at either 2 h or 24 h of incubation. These results demonstrate that iodoquinol displays low cytotoxicity toward A549 cells, even at concentrations substantially higher than those required for antifungal activity.

## DISCUSSION

Opportunistic fungal infections have been increasingly reported since the 1990s, whose pathogens have been presenting high virulence and resistance to antifungal drugs (1, 6, 7). Many efforts have been observed in the literature to investigate molecules with promising antifungal activity. A previous study from our group has demonstrated that auranofin and iodoquinol are active against *Scedosporium* and *Lomentospora* species (13), but little is known about their effects on fungal cells, as well as their activity against other fungi mentioned on the WHO List of Priority Fungal Pathogens, such as *Aspergillus*, *Fusarium,* and Mucorales species.

In the present work, auranofin was more active against *A. fumigatus*, *A. flavus*, *S. boydii*, *L. prolificans,* and *F. oxysporum*, whose MIC values ranged from 5 to 10 µM. Mucorales species revealed more tolerance to auranofin, whose MIC varied between 20 and >40 µM. These data are in accordance with other studies, which demonstrated that auranofin MIC ranged from 1 to 16 µg/mL (1.5–24 µM) against *A. fumigatus*, *S. apiospermum,* and *L. prolificans* (13, 28). Fungicidal effect of auranofin ranged between 10 and >40 µM, being more active against *S. boydii* and *A. fumigatus*. Fungicidal activity of auranofin has also been shown against *Aspergillus* species and *Candida albicans*, but a fungistatic effect has been demonstrated against *Cryptococcus neoformans* (39, 40).

A similar activity was observed for iodoquinol, which was more active against *A. fumigatus*, *A. flavus*, *S. boydii*, *L. prolificans,* and *F. oxysporum*, whose MIC values ranged from 0.65 to 5 µM, and less active against Mucorales species, which were inhibited with iodoquinol concentrations of at least 20 µM. Antifungal effects of iodoquinol have already been demonstrated against other fungal pathogens, such as *Sporothrix*, *Phialophora*, *Fonsecaea*, *Exophiala*, *Candida*, *Scedosporium,* and *Lomentospora* species, ranging from 5 to 10 µM (12, 13). On the other hand, the present study revealed that iodoquinol displays fungistatic activity against all fungi. Coelho and colleagues also showed a fungistatic effect of iodoquinol against *C. carrionii*, *E. dermatitidis*, *E. jeanselmei*, *F. pedrosoi*, *F. nubica,* and *R. similis*, but fungicidal activity has been observed against *P. verrucosa* and *F. monophora* (10).

Kinetic assay revealed that both auranofin and iodoquinol could inhibit fungal growth even at early stages, because a difference in fungal density is seen from 12 h of incubation. It is the first time that kinetic studies are performed to investigate auranofin and iodoquinol.

Fungal biofilms are relevant structures able to increase the severity of infections and the resistance rates to antifungal drugs. In the present study, both auranofin and iodoquinol reduced biomass and biofilm viability of all fungi tested. Anti-biofilm activity of auranofin has already been described in *Candida*, *Aspergillus*, *Scedosporium*, and *Lomentospora* species, as well as in mixed biofilms formed by *C. albicans* and *Staphylococcus aureus* on catheter surface (13, 29, 40, 41). Besides reducing the biofilm formation on abiotic surfaces, auranofin decreases bloodstream infection caused by contaminated catheters in a murine model (29). Regarding iodoquinol, its activity against fungal biofilms has already been demonstrated in *Scedosporium* and *Lomentospora* species in our previous study and in *C. albicans* (12, 13). These data evidence the promising effect of both auranofin and iodoquinol against a known resistant structure of pathogenic fungi.

Aiming to analyze some cellular alterations caused by auranofin and iodoquinol against pathogenic fungi, cellular stressors and fluorescent staining were used to check some cell alterations in the presence of both compounds. Auranofin treatment led to an increased susceptibility to SDS in most fungi, as well as a reduction in Nile Red staining, suggesting that auranofin affects fungal lipids and plasma membrane. It has already been demonstrated that auranofin partially inhibits the lipid biosynthesis of *S. aureus* (41). In addition, it enhanced the susceptibility of *S. boydii*, *R. oryzae,* and *M. velutinosus* to NaCl, as well as decreased the concanavalin A and calcofluor staining in *A. fumigatus*, *L. prolificans,* and Mucorales species, suggesting the occurrence of cell wall alterations. It has already been reported that auranofin presents a wide spectrum of action and can affect the biosynthesis of cell wall and plasma membrane components (41, 42).

Auranofin also caused an increase in susceptibility to menadione for all fungi, corroborating its mechanism of action, which is based on the inhibition of the enzyme thioredoxin reductase (TrxR). Some studies have demonstrated that auranofin presents activity against TrxR from a variety of microorganisms, such as *Trichomonas vaginalis* and *Aspergillus* species, impairing gene expression and enzymatic activity of TrxR (40, 43). In addition, TrxRs are crucial for *Saccharomyces*, *Cryptococcus,* and *Aspergillus* species to be protected against ROS stress (14, 40). In *Scedosporium* and *Lomentospora* species, it has already been shown that the addition of menadione enhances susceptibility to auranofin, suggesting that it impairs fungal protection against oxidative stress (44).

With respect to iodoquinol, it increased the susceptibility of *A. fumigatus*, *F. oxysporum*, *R. oryzae,* and *M. velutinosus* to SDS and NaCl, as well as reduced Nile Red staining in most fungi tested. In addition, iodoquinol also affects the susceptibility of some fungal species to NaCl and menadione, as well as the concanavalin A and calcofluor staining. However, only a few studies in the literature have shown the effects of iodoquinol on fungal cells. *Sporothrix* species treated with iodoquinol displayed plasma membrane disruption and leakage of intracellular content (9). In *Candida* species, iodoquinol inhibits pseudo-hyphae development and compromises the integrity and permeability of the plasma membrane (36). These data suggest that iodoquinol could present different targets on fungal cells and interfere with the stability of the fungal cell surface.

Regarding the electron microscopy images, the treatment of *F. oxysporum* with auranofin or iodoquinol resulted mainly in a reduction of hyphae length, as well as in extracellular leakage. Treatment of *F. oxysporum* with other compounds also causes the leakage of cellular content, as demonstrated with miltefosine (45). However, SEM data were more interesting with *A. fumigatus*, which revealed that it secreted a significant amount of extracellular polymeric substances when treated with auranofin or iodoquinol. Some compensatory mechanisms for antifungal resistance have already been reported. The use of voriconazole against *A. fumigatus* resulted in a thicker cell wall, which suggests that the synthesis and secretion of cell wall components is an important attempt to resist antifungal action (46). In *Scedosporium* species, treatment with miltefosine also led to an increase in cell wall thickness (19). Interestingly, our data using concanavalin A and calcofluor staining did not show an increase in the polysaccharide detection on fungal surface, which could be controversial with electron microscopy analysis at first sight. However, the fungal growth conditions were different between these assays, which could explain the differences found in these data. It is already known that *A. fumigatus* presents galactosaminogalactan on the cell surface, which is important for fungal virulence, adherence, and biofilm formation (47–49). The secretion of these types of molecules could be a compensatory mechanism to protect fungal cells against the stress caused by auranofin and iodoquinol, but the identification of these polymeric substances observed in SEM images remains unknown, and more studies are needed to elucidate which molecules are being secreted by these fungi when they are treated with a sub-inhibitory concentration of auranofin and iodoquinol.

The strategy of combined therapy is interesting to avoid the emergence of resistant strains and to improve the treatment of fungal infections. Using FICI and BLISS methods, auranofin and iodoquinol presented synergistic or additive effects with voriconazole and amphotericin B, but not with posaconazole, as observed with *A. fumigatus*, *F. oxysporum*, *L. prolificans,* and *R. oryzae*. The study of the interaction between auranofin or iodoquinol with antifungal agents is rare in the literature. Rollin-Pinheiro and colleagues have demonstrated that both auranofin and iodoquinol display a synergistic effect with caspofungin in the model of *Scedosporium* and *Lomentospora* species, but no effect has been observed with voriconazole (13). In addition, Coelho and colleagues have shown that the interaction between iodoquinol and itraconazole is indifferent in the model of chromoblastomycosis agents (10). These data suggest that the pattern of combination between auranofin or iodoquinol with antifungal agents varies among fungal pathogens, and more studies are needed to elucidate the potential interactions between these compounds.

When analyzed together with antifungal susceptibility data, cytotoxicity results in A549 cells clarify the therapeutic potential of both compounds. Auranofin showed antifungal MICs of 5 to >40 µM, overlapping with time- and dose-dependent cytotoxicity in A549 cells, consistent with reports of low-micromolar IC_50_/CC_50_ values in mammalian cells due to inhibition of thioredoxin reductase and redox imbalance (39, 50–52). Despite this overlap, its clinical use suggests that *in vivo* tolerability is influenced by pharmacokinetics and exposure duration rather than static *in vitro* thresholds (53). In contrast, iodoquinol exhibited a more favorable profile, with antifungal MICs (0.625–40 µM) often occurring below cytotoxic concentrations in A549 cells. This agrees with studies reporting sub- to low-micromolar MICs and CC_50_ values ≥5–25 µM, yielding a measurable selectivity window (9, 13). Mechanistically, its antifungal activity is linked to metal chelation and disruption of fungal homeostasis, which may underlie its lower mammalian cytotoxicity (54). Overall, while auranofin’s antifungal activity may be limited by cytotoxicity at higher concentrations, iodoquinol displays a broader *in vitro* therapeutic margin. However, the cytotoxicity profiles of both drugs and their prior clinical use indicate that they are safe for use in humans. Reported side effects of auranofin and iodoquinol are primarily associated with prolonged treatment and high doses, respectively (55, 56).

Taken together, all these data presented in the study indicate that auranofin and iodoquinol are promising compounds to be developed and used against fungal infections, and further work is needed to guide new studies in this direction.
